# Retraction: Optimization of diosgenin extraction from *Dioscorea deltoidea* tubers using response surface methodology and artificial neural network modelling

**DOI:** 10.1371/journal.pone.0293108

**Published:** 2023-10-12

**Authors:** The PLOS ONE Editors

Following the publication of this article [1], concerns were raised regarding the results presented in Fig 2. Specifically,

- In Fig 2A, the following lanes appear similar:
  ○ Lanes 1–4 and lanes 16–19
  ○ Lanes 5, 6, and 20
  ○ Lanes 7 and 21
  ○ Lanes 8–10, lanes 12–14, and lanes 22–24
  ○ Lanes 11 and 25
- Undisclosed conflicts of interest were identified involving the authors, Academic Editor, and reviewers that call into question the integrity and objectivity of the article’s peer review.

The corresponding author explained that the wrong set of photographs were inadvertently used during the preparation of Fig 2A and provided a replacement figure. However, the corresponding author’s explanation does not resolve the concerns raised with the published figure.

In light of these issues, the *PLOS ONE* Editors retract this article.

TM did not agree with the retraction. RN, DKP, BP, VK, PD, AK, and AD either did not respond directly or could not be reached.
